# Exploring a New Model of End-of-Life Care for Older People That Operates in the Space Between the Life World and the Healthcare System: A Qualitative Case Study

**DOI:** 10.15171/ijhpm.2019.138

**Published:** 2020-01-04

**Authors:** Steven Dodd, Nancy Preston, Sheila Payne, Catherine Walshe

**Affiliations:** International Observatory on End of Life Care, Division of Health Research, Lancaster University, Lancaster, UK.

**Keywords:** Health Services, Frail Elderly, Palliative Care, Aged, Qualitative Research

## Abstract

**Background:** Innovative service models to facilitate end-of-life care for older people may be required to enable and bolster networks of care. The aim of this study was to understand how and why a new charitably funded service model of end-of-life care impacts upon the lives of older people.

**Methods:** A multiple exploratory qualitative case study research strategy. Cases were 3 sites providing a new end-of-life service model for older people. The services were provided in community settings, primarily providing support in peoples own homes. Study participants included the older people receiving the end-of-life care service, their informal carers, staff providing care within the service and other stakeholders. Data collection included individual interviews with older people and informal carers at 2 time points, focus group interviews with staff and local stakeholders, non-participant observation of meetings, and a final cross-case deliberative panel discussion workshop. Framework analysis facilitated analysis within and across cases.

**Results:** Twenty-three service users and 5 informal carers participated in individual interviews across the cases. Two focus groups were held with an additional 12 participants, and 19 people attended the deliberative panel workshop. Important elements contributing to the experience and impacts of the service included organisation, where services felt they were ‘outsiders,’ the focus of the services and their flexible approach; and the impacts particularly in enriching relationships and improving mental health.

**Conclusion:** These end-of-life care service models operated in a space between the healthcare system and the person’s life world. This meant there could be ambiguity around their services, where they occupied a liminal, but important, space. These services are potentially important to older people, but should not be overly constrained or they may lose the very flexibility that enables them to have impact.

*“Building a workforce that can meet this population’s needs will require more than training … it will require a radical redesign of the health system that is centered on the communities where patients live.”*

Jessica Bylander ^1^

At the *Health Affairs* summit in 2018, the need to redesign healthcare for those with serious illness was clearly set out.^1^ Many palliative care services are found mostly in hospitals, and hospice care may only be offered to those imminently dying.^2-4^ There is a need to develop palliative care service models to meet the needs of those, often older people, living in the community who are frail and with co-morbid conditions, and who are likely to be towards the end of their lives.^5^ In the context of this study we use the term ‘approaching the end of life,’ meaning likely to die within the next 12 months. This includes people with advanced, progressive, incurable conditions, general frailty, and coexisting conditions that mean they are expected to die within 12 months.^6^

An important element of care provision towards the end of life may be based within and from the community, provided via social networks and using people’s social capital.^7,8^ Social relationships and networks can buffer distress or crisis situations, prevent family carer burn out, and demonstrate the importance of social contexts.^9,10^ However, existing networks can be small and fragile, community engagement reduced by existing caregiving responsibilities, and with formal care services providing little practical support.^9^ Older people may have smaller social networks, and family carers themselves may be older. Compared to other caregivers, end-of-life caregivers provide nearly twice as many hours of care per week and, report more care-related challenges.^11^ Social isolation of itself also has a major influence on health, comparable with well-established risk factors for mortality.^12^ For older people in particular, innovative solutions and service models may be required that enable and bolster networks of care.

Service models to facilitate access to healthcare for frail older people include care coordination, case management, care navigation, and integrated care, with variable effects on outcomes such as satisfaction, health status, healthcare utilisation or place of death.^13-19^ What these interventions share is their mediation by health or social care professionals working within formal care networks, often with a relatively narrow focus. It may be that service models that sit outside these formal care networks could offer a flexible, innovative, community focused solutions to improving access to care for those who may not usually access palliative or hospice care services, meet needs and enable improved quality of life.

In this paper research is presented exploring the impact of a new service model of care towards the last years of life for older people. A UK charity focused on older people (Age UK) set up 3 pilot services facilitating care to older people thought to be in their last 12-18 months of life. Sitting outside the formal healthcare system, the service model involved a number of aspects. First, encouraging referral of those thought to be in their last 12-18 months of life primarily through working alongside general (family) practitioners and publicity to local health and social care providers. Second, training staff (not necessarily with a health or social care background) to enable conversations with older people and facilitate integrated support to achieve personal goals. Third, mobilising volunteers to provide support where required. Such services were provided alongside any existing care. The aim of this research was to understand how and why this new charitably provided community model of end-of-life care influences the experiences of older people.

## Methods

### Research Design

We conducted a multiple exploratory longitudinal qualitative case study research strategy.^20,21^ As the services were new, a longitudinal design enabled tracking both service development over time (6 months), and any changing impacts from those using the service. The case was defined as a location providing the new model of care and those involved with commissioning, referring to, delivering and receiving the service.

### Case Selection and Setting

Three locations piloted the new service model, and these formed the 3 cases for the research. Each served a different geographical area. Brief information on the geographical area each served is given in Table 1 to contextualise the cases for the reader.

**Table 1:** Contextual Information on the 3 Locations Piloting the New Service Model

**Site**	**Description of Location**	**Ethnicity of Location** ^a^	**Older Person Population** ^a^	**Mortality Rate by Age Group** ^b^	**Place of Death 2016 All Ages** ^b^	**Referral Sources for New Service Model**
1	Market town Population 20-30 000	White 97.9%	60-64: 7.4% 65-74: 11% 75-84: 6.6% 85-89: 1.7% ≥90: 0.9%	0-64 years: 11.5% 65-75 years: 18.7% 75-84 years: 28.2% 85 years+: 41.6%	Hospital: 50.4% Care home: 23% Home: 20.9%	1 Family practice (general practitioner surgery)
2	Suburban, part of large urban (Population 320 000) area	White 97%	60-64: 6.8% 65-74: 9.8% 75-84: 6.7% 85-89: 1.7% ≥90: 0.9%	0-64 years: 15.2% 65-75 years: 16.1% 75-84 years: 29.3% 85 years+: 39.3%	Hospital: 50% Care home: 20.8% Home: 23.8%	2 Family practices (general practitioner surgeries)
3	Mid-size town Population 200-300 000	White 84.5%	60-64: 5.5% 65-74: 7.0% 75-84: 4.4% 85-89: 1.3% ≥90: 0.7%	0-64 years: 17.6% 65-75 years: 17.6% 75-84 years: 25% 85 years+: 39.8%	Hospital: 51.5% Care home: 18.8% Home: 21.7%	Varied number of referral routes

^a^ Census Data; ^b^ Data from Public Health England: end of life care profiles.

### Participants

Participants included older people receiving care from the new service model, their family carers, service providers and other stakeholders including general practitioners, community nurses, and Age UK charity staff. A broad definition of ‘family carer’ was used, including those related through committed heterosexual or same sex partnerships, birth or adoption and others who have strong emotional and social bonds with the service user. They are lay, unpaid, people in a close supportive role who share in the illness experience of the service user. Inclusion and exclusion criteria for older people and family carers are specified in Table 2. Staff and stakeholders were included if they were involved in providing the service in the selected locality including as a volunteer or manager, or were associated with the service in the selected locality in a stakeholder role including local commissioners and policy-makers, providers of health and social care service(s) to older people in the locality, or other locally identified stakeholders.

**Table 2:** Inclusion and Exclusion Criteria

**Inclusion Criteria**	**Exclusion Criteria**
**Older Person Inclusion and Exclusion Criteria**	
Receiving the service in the selected locality.	Lack capacity to consent to participate in the research, as assessed by site staff or involved clinicians.
Aged ≥50 years, no maximum age. This age was set by Age UK as the minimum age to receive their services.	Unable to participate in a qualitative interview using English, as assessed by site staff.
**Family Carers (Including Bereaved Carers ) Inclusion and Exclusion Criteria**	
Identified (by the older person) as the family carer of an older person receiving (or who has received, in the case of bereaved carers) the service in the selected locality.	Lack capacity to consent to participate in the research, as assessed by site staff or the person taking consent.
Aged ≥18 years, no maximum age.	Unable to participate in a qualitative interview using English, as assessed by site staff or the person taking consent.
	For bereaved carers, those who Age UK staff identify has having adverse, complex, or prolonged grief reactions following the death of the person they cared for.

### Sample

A convenience sample of older people referred to, and receiving care from, the service in each location was obtained, and if available, their family carer. A sample size of up to 10 older people and 5 family carers was anticipated per case. All those providing direct care as part of the new service model, and a purposive sample of stakeholders to include a range of people from different professional backgrounds with an interest in the service, were invited to participate.

### Recruitment

Those providing the service distributed recruitment packs (invitation letter, participant information sheet, reply slip). Older people who indicated interest were contacted to arrange a face-to-face visit, where written consent was obtained. Older people were asked to pass a recruitment pack to a family carer of their choice. Staff and stakeholder participants received information about the study either directly from the research team, or via the service managers.

### Data Collection

Six forms of data collection were used:

1. *Individual interviews* : Older people and/or their family carers were interviewed to explore their experience of receiving the service. Initial interviews were face to face, with follow up telephone interviews offered approximately 3-4 months later. Demographic data were collected at the first interview. All interviews were conducted by SD or CW, digitally audio-recorded and transcribed.
2. *One-off focus group interviews* in each case study site with staff and stakeholders to explore views on the service. These were conducted by CW or SD and digitally audio-recorded and transcribed, and details only of the roles of participants collected.
3. *Non-participant observation* of a service or other relevant meeting within each case study site were conducted by SD and facilitated an understanding of service processes.
4. *Non-patient specific documentary materials such as service brochures* were collected from each case-study site to understand how the service was conceptualised and publicised, referral and other service processes.
5. *Service provision data* : Anonymised aggregated data were provided by the sites summarising demographic information on all referrals received.
6. *Deliberative panel discussion workshop:* A cross-case deliberative panel was held with staff key workers and stakeholders, together with selected professional and lay experts. This consisted of brief presentations of core findings followed by small group facilitated discussions. The panel was facilitated by CW, NP, and SP. The purpose of the deliberative panel workshop was to discuss the emerging findings of the case studies, to facilitate interpretation and identify key themes.

A topic guide for the individual and group interviews, and the deliberative panel, can be found in Supplementary file 1.

### Data Analysis

Framework analysis, used in previous palliative care case studies and which facilitates integration of different forms of data, was used to facilitate within and cross case pattern matching.^21-23^ The approach involves a systematic 5-stage process of familiarisation, identifying a thematic framework, indexing, charting, and mapping and interpretation. An a priori thematic framework^22^ was informed by 4 theoretical propositions developed from an initial scoping review of qualitative literature examining outcomes of services for older people with similar features:

1. Enriching relationships: Relationships engendered feelings of being more cared for, respected, loved, and secure.
2. Greater autonomy and perceived control: People felt more empowered, understood, consulted.
3. Knowing more: More involved and informed. The intervention promoted a greater level of engagement and knowledge in the patient about their condition.
4. Improved mental health: People felt less anxious or stressed. They could also be more confident, more independent and more assertive.

This framework iteratively developed throughout the analysis. Analysis was primarily conducted by SD (health services researcher), with cross checking and agreement of coding with CW (researcher with palliative care nursing background), and discussion with NP and SP (researchers with nursing and psychology backgrounds) to debate areas of disagreement. Cross case pattern matching follows to identify thematic factors associated with challenges and successes in influencing the experience of older people whilst taking account of context. All qualitative analyses were managed using NVivo™ software.

## Results

Twenty-three service users and 5 informal carers participated across the cases, their demographic information, and that of all referrals received are found in Table 3.

**Table 3:** Demographic Information on All Referrals to the Services From Inception to End of Data Collection Period, and of Interview Participants in the Study

**Service Users**	**Site One**		**Site Two**		**S ite Three**	
	**Referred to Service**	**Study Participants** **(n = 10)**	**Referred to Service**	**Study Participants** **(n = 6)**	**Referred to Service**	**S tudy Participants** **(n = 7)**
Number referred	74	NA	102	NA	23	NA
Time period for referral receipt	14 months		14 months		8 months	
Mean age (range)	81 (52-100)	82 (67-97)	86 (44-97)	89 (82-93)	80 (56 to 93)	80 (67-86)
Male	30	2	36	4	6	1
Female	44	8	65	2	17	6
Missing data	0	0	1	0	0	0
Married	Data not recorded	2	Data not recorded	1	Data not recorded	1
Divorced		1		0		2
Widowed		7		4		2
Not disclosed		0		1		2
Live alone	Data not recorded	7	Data not recorded	4	Data not recorded	5
White British	69	8	71	5	5	6
White other	2	0	0	0	2	1
Black Caribbean	0	0	0	1	1	0
East Asian	3	0	1	0	0	0
Missing data	0	2	30	0	15	0
*Primary diagnosis*						
Cancer	11	1	12	0	Data not recorded	1
Respiratory	14	3	6	0		2
Cardiac	6	2	9	1		1
Neurological	7	1	3	1		0
Dementia	3	2	2	0		0
Frailty	0	1	12	0		0
Musculoskeletal	6	0	6	1		0
Other	9	0	12	0		0
Missing data	18	0	40	3		3
Mean number diagnoses (range)	3.5 (1-7)	2.9 (1-5)	2 (1-2)	1(1-3)	Data not recorded	2 (1-3)
Informal carers						
Number		3		1		1
Relationship to service user		Spouse; carer; friend		Son		Spouse

NA, not applicable.

In site 1 follow up interviews were conducted with 4 service users and one informal carer and in site 2 with 2 service users but were not possible in site 3 due to deterioration or death of participants. Initial interviews lasted a mean of 26.3 minutes (range 8.4–45.3). The 2 focus groups lasted 56.3 and 71.1 minutes. The focus group in site 1 had 7 participants (3 provider staff, 4 external stakeholders), and in site 3 had 5 participants, all provider staff. A full day deliberative panel workshop included a number of different discursive elements across the day. Participants for the deliberative panel are summarised in Table 4.

**Table 4:** Participants in the Deliberative Panel

**Participants in Deliberative Panel**	**Number**
Staff from service funders headquarters	n = 5
End-of-life care service site staff	Site 3 n = 2 Site 2 n = 3 Site 1 n = 2
NHS representatives	Site 2 n = 1 Site 1 n = 2
Lay representative	n = 1
Researchers	n = 3
Total	19

Abbreviation: NHS, National Health Service.

The cross-case analysis is presented here, as the 3 overarching themes identified from the analysis. First, organisational identity, exploring how and why the services conceptualised and organised themselves, and how they fitted with existing service provision structures. Second, flexible provision, how and why services were provided, their focus, and the type of care offered. Third, the impact and experience of the service, how people experienced the services, and what the impacts of the service were for users.

### Service Organisational Identity

These services were often perceived as ‘outsider’ services, although this was not clear-cut:

*“I think we are insiders, in that we are a community service, so we are maybe within the community voluntary sectors providing a community service, but I suppose outsiders in terms of we are not health professionals”* (Deliberative panel - staff).

Being ‘outsiders,’ compared to health and social care staff, could be perceived as an advantage:

*Speaker 1: “the plus about us is, we are outsiders…we have a staffing that is more fluid (…) and that fluidity is what is noticed very much by statutory services isn’t it.”*

*Speaker 2: “The flexibility we bring is exactly the opposite of an institutional approach and, of course, our workers will do whatever”* (Site 3 Focus group - staff).

The service staff also often regarded themselves as outsiders, and whilst this could be a source of frustration in gaining credence with and access to healthcare providers (such as being able to attend healthcare meetings, and the challenges of insufficient initial referrals), they recognised that this enabled them to take risks, that would not be allowed by public service professionals because of bureaucratic processes:

*“We go, ‘oh come on,’ and we just get it done…, risks work, they work very much in favour of the clients, (…) we will do those things that can’t be done under that red tape”* (Deliberative panel - staff).

*“You walk into someone’s house and you know, they’ve got no food because they haven’t got a fridge, and you know, you’re the only person that’s going to see them for the next week, as an organisation we’ll go and get them a fridge, and we’ll carry the fridge into their house and we’ll plug the fridge in. But, you know, I think of the millions of risks attached to…”* (Deliberative panel - staff).

‘Risk taking’ was almost universally perceived to be of benefit to clients. Typical risks were unlikely to pose direct harm, but which, as in the examples above, circumvented ‘red tape’ to meet expressed needs directly and rapidly in the way that a friend or neighbour might do.

The predicament of being an outsider was that it could be hard to establish credibility and form a clear identity for the service. This could make it more difficult to gain a sympathetic audience with potential referrers, particularly when the purpose of more flexibly provided services may not be clear to them. The services struggled initially to gain referrals, and recognised that their planned associations just with general practices needed to be widened.

### Flexible Provision

The case study sites differed in the structure of their approach, within the overall initially proposed service model. For example, site one had a more structured approach, restricting their role to a narrower range of predefined tasks such as future care planning and assistance with benefit applications. Whereas, in site 3, more flexibility and autonomy could be seen in how they interpreted their role and what to do with service users, for example in providing more direct befriending services and a ‘listening ear.’ In site 2 their service had developed away from a fixed approach toward becoming more flexible in their response to need:

*“I think we started off with quite fixed criteria and within a very short time we realised it’s not going to work, and you do have to become more flexible, don’t you, and the things that you were perhaps thinking, like we were saying about advance care planning and power of attorney…you thought you would be really focusing on those”* (Deliberative Panel staff member).

Flexibility however, resulted in challenges expressing their service identity or purpose, despite the benefits of responsiveness or addressing unanticipated needs:

*“I think the holistic thing is important in this role, because if that…I’ve got one client that I’ve done, I think, seven different things for him and that varies from maximising his income, referral to occupational therapy, getting him some rehabilitation at home, getting him out and socialising ”* (Deliberative panel - staff).

Lacking a clear, defined, identity and purpose was confusing both to referrers, affecting referral streams, and to service users initially who could be confused about the referral and its purpose.

### Service Impacts and Experiences

#### Enriching Relationships

The relational aspects of care, and human contact, appear important. Service users expressed satisfaction at being party to a new relationship, in some case likening it to a friendship, providing much needed company and contact with the outside world. The relationship in and of itself could be experienced as a profound impact of the intervention:

Interviewer: “What would you say have been the biggest benefits you’ve felt from the service?”

*Service User: “Just knowing them. Such satisfaction of knowing these people”* (Interview S1P4).

The depth of the relationship could differ, but it was common for service users to emphasise their appreciation for the relationship, irrespective of how formal or ‘loose’ the relationship was:

*“It’s important for the likes of me to have that lovely regular but loose connection, a little bit of a chat, a little bit of support, little bit of understanding. A lot of understanding and to help where necessary, yeah?”* (Interview S2P6).

Service staff felt that it could take time to build up relationships to enable in-depth discussions, but that this was not always possible when someone had a limited prognosis:

*“Basically because of capacity, that the time it takes to do that kind of work and the relationship…the time it takes to build up the relationship to have that kind of conversation about end of life, really”* (Site 1 Focus Group - staff).

Despite this, service user’s trust for the service staff could reach a level where they felt that they could act like a confidante for them, facilitating frank conversations that the service user might feel uncomfortable having with friends or family:

*“I didn’t know who to turn to, or anything, but now I’ve got someone I can turn to that I know I can… you know, you don’t mind confiding in”* (Interview S2P4).

Such relationships could affect people’s state of mind:

*“The most important thing is knowing that there’s somebody there who you can contact if you’re unsure of any difficulties, and if they can’t give you the answer, they know somebody who can….. And I think that’s very important that you know that there’s somebody out there, you know, who can”* (Interview S1P7).

#### Improved Mental Health

Service users appeared to derive psychological benefits from their relationships with the service staff:

*“If you’d come before she started coming, you would have noticed a difference in me, you know. I just didn’t want to talk to people and, you know. It’s only a few visits, but it’s so much” (Interview S3P2).*

Others made explicit references to impacts such as alleviation from worries or anxiety:

*“Very aware that, obviously, I’ve problems and worries and things, so she put my mind at rest about a lot of those so I didn’t feel at all intimidated by her coming in to talk to me or asking me questions”* (Interview S1P3).

*“Keeping me chirpy and not going down that pit of anxiety, she’s there. She’s there. Yeah. (….) It’s an extra, it’s an extra part of being comfortable with who I am and what I can do and what I can’t do”* (Interview S2P6).

Service users also mentioned greater feelings of safety and security:

*“It takes pressure off you because you feel… I know this sounds daft, somebody my age, but you feel safer somehow and that’s a big thing”* (Interview S1P7).

In more vivid terms, this service user described the feelings of protection and security he felt:

*“I’m not standing on the end of a cliff feeling like I’m going to fall, you know what I mean? And they come and help, it’s like having a barrier and they put a blanket round you and cuddle you”* (Interview S1P9).

#### Financial Impacts

Service staff frequently supported service users to apply for financial benefits, making up a considerable shortfall in the service user’s finances:

*“It [new financial benefit] will make a big difference to me, yeah, (…) That’ll pay the carers , but then I’ve got to live on my savings”* (Interview S3P5).

#### Being Part of the Outside World

For some individuals the company of the service staff or volunteers could constitute a rare instance of social contact, and where they could be enabled to leave their homes and be part of a world they had lost:

*“I’m so used to not being out for so long, you know, that it’s a treat for me to sit here and think, you know. Well, when she asks me another time, you know, one day, ‘What shall we do?’ I shall say, ‘Well, let’s go around and have a look at the new café,’ It doesn’t take much to please me if I go out”* (Interview S3P2).

## Discussion

### Summary of Main Findings

The services occupied a distinct space in their local care landscape in providing a different, but needed, form of care to older people towards the end of their lives. They identified challenges articulating and defining the form of provision, and this resulted in subtle differences in service scope, form of provision, and the degree of responsiveness and flexibility. Services were perceived to be ‘outside’ the norm of service provision, but this enabled them to take more risks, responding to need in ways not possible for traditional service providers. Service users reflected this ambiguity, with some lack of clarity about the purpose of the service. However, where a relationship developed, this was described as having a needed impact on feelings of having a friend, on being part of, or re-engaging with, a community, and having someone to turn to. There were impacts described on mental health issues such as general worries, anxiety and depression, with people feeling safer and more secure.

### What This Adds to Knowledge

These services operate in a space that can be understood with reference to Habermas’s description of system and lifeworld.^24,25^ Habermas argues that as social complexity increases, our economic and political systems become disconnected from the personal or family lifeworld. These services could be seen as operating in a space between the (healthcare) system and the (personal) lifeworld, where there could be discomfort or conflict if they bring the attitudes, values and needs of the patient’s lifeworld into a rule-bound and risk averse healthcare system. Occupying these spaces can be experienced as ambiguous, where the social expectations that may be between, say a nurse and a patient, are suspended, making this a ‘liminal’ or ‘threshold’ space. Service users appeared to recognise these services occupied a different form of space, with different expectations than of formal care services.

These concepts of system and lifeworld have been used to explore issues such as hospice provision, community nursing and public involvement, where there is also perceived to be a space, or shift in lifeworld.^26-28^ The concept of liminality can both conceptualise the ‘betwixt and between’ nature of the space between living and dying or where serious illness alters a certain lifeworld,^29,30^ and the flexible services that can operate between system and lifeworlds.^31^ Liminality expresses how they existed in the interstices between categories of insider/outsider, inhabiting characteristics of being an outsider such as being risk-taking, flexible, and exerting affective labour, while, simultaneously, exhibiting characteristics of insider status such as being paid workers for a well-known charity, gaining access to service users through family practitioners, and having a degree of expertise in their field. There was a degree of agency and choice at work in how each service choose to work with liminality and interpret the identity of their service, pushing their working practices towards structure or flexibility, insider or outsider, and risk takers or risk averse services.

Engendering a feeling of safety, security and being cared for was also important to the experience of receiving the service. Feeling safe is emerging as a core concept in many healthcare decision-making processes, including decision-making about going to emergency departments^32^ or being in hospital.^33^ There is evidence that home nursing services can enable a feeling of safety,^34^ and that if neighbors are trusted, that engenders a feeling of safety that improves self-reported health.^35^ Feeling safe and secure appears to protect against frailty.^36^ Whilst we report that older people reported benefit from the relationship itself, rather than a specific impact of the relationship, there is strong evidence that social relationships, loneliness and social isolation affect mortality risk.^12,37^ Whilst the ‘lifeworld’ places importance on people and relationships, it is likely that this impact on health and well-being could influence use of healthcare ‘systems.’

### Strengths and Limitations of the Research

The case study approach enabled a multi-perspective understanding of how and why these new service models had an impact on service user experience. However, we captured little of the family carer view on these services and their impact, and it may be that family carers have different opinions on services and their impact. Information about the study was given to service users by the services, due to requirements of our research approvals. They may have selected potential participants in unknown ways that introduces bias, for example those who may have expressed positive opinions of services received. We do not know how many were given information packs but chose not to participate. Our sample was primarily White British, which reflects the users of these services, and typical users of many end-of-life care services.^38^ Access to palliative care services for minority ethnic populations remains challenging,^39^ and despite one case study being situated in an area with a considerable minority ethnic population, it appears these services may not be the answer to addressing this inequitable access. It was challenging to capture repeated interviews, primarily due to health deterioration or death, and the planned longitudinal understanding is not present in this analysis. Only 2 of our initial 4 theoretical propositions were fully supported by the data; those of enriching relationships and improved mental health. Our initial scoping review drew from a number of studies on advance care planning, given this was planned to be a focus of the intervention studied. However, such planning conversations were not a key part of the service model in some locations, and it may be this is why propositions on autonomy and knowledge were not supported by these data.

### Recommendations for Policy, Practice and Future Research

Policy-makers and practitioners should consider facilitating or initiating such services as they appear to have value. Account needs to be taken of ways of enabling sufficient time to allow flexibility and reasonable risk taking that appear vital to success, even if referrals and service usage increases. Evaluation should be integral, taking account of how contexts shape such services, and consideration should be given to attributional and/or longitudinal designs to strengthen the evidence base and enable appraisal of service outcomes such as on quality of life.

## Conclusions and Implications

Flexible, responsive, person-centred services, operating in the liminal space between the person’s life world and formal health and care systems, appear to have benefit for older people thought to be towards the end of their lives. The benefit is likely to be in aspects such as developing relationships, feeling connected and safe, and well-being. These benefits may have an impact on mental health, mortality and service use.

## Ethical issues

North-West Liverpool East Research Ethics Committee granted approval on February 2, 2018: 17/NW/0705.

## Competing interests

Authors declare that they have no competing interests.

## Authors’ contributions

CW, NP, and SP secured the funding to conduct this work and were responsible for conceptualising the study. SD and CW collected and analysed data. SD drafted the initial manuscript, and all authors are responsible for critical revisions and approval of final manuscript.

## Funding

This work was supported by Age UK.

## Supplementary files


Supplementary file 1. Data collection topic guides and schedules.

## Key messages

**Implications for policy makers**

- Flexible service models that are provided without formal, statutory health and social care funding may offer particular and specific benefits to older people towards the end of their lives.
- Service models provided outside of usual care provision should be enabled to be flexible, responsive and risk taking to facilitate a different sort of impact on older people.
- Articulating the roles a new service model may provide is important, but there should be the facility for this to change in response to actual patient need.
- Supportive end-of-life care service models operating in a community or voluntary provided space are likely to have an impact on enriching relationships and improving mental health.

**Implications for the public**

Older people who live at home towards the end of their lives may have care and support needs that are not met by traditional health and social care services such as nurses, doctors or social care services. We found that a new, charitably provided, service model focused on responsive identification of care needs, service referral and befriending operated in an important space between formal care services and the support of friends and family. They appeared to help people to feel safe and secure, re-connect them with their communities, and enable improvements in people’s mental health. Service providers could consider how they may replicate such care models within their own contexts.
