# Supplementary material for: Exploring a New Model of End-of-Life Care for Older People That Operates in the Space Between the Life World and the Healthcare System: A Qualitative Case Study
Source: Int J Health Policy Manag. 2020 Jan 4;9(8):344–51. doi: 10.15171/ijhpm.2019.138 (PMC7500390; doi:10.15171/ijhpm.2019.138)
Supplement: Supplementary file 1 — Data collection topic guides and schedules. [file ijhpm-9-344-s001.pdf]

Supplementary file 1

Data collection topic guides and schedules

1) *Individual interviews with service users:*

A topic guide rather than a fixed schedule guided but not constrained the interviews, ensuring that interviews were driven by participant issues. Interviews were conversational to aid developing rapport to explore complex and potentially emotional issues. The topic guide evolved as categories are discovered through the interviews and analysis. Interviews were terminated at participant request or if the interviewer is concerned about the participant.

Interview topics:

a) Summarising experiences as patient/carer to set agenda, level of disclosure and terminology.
b) Probing events i.e. could you tell me a bit more about what happened then? How do you feel about that? Did you get support from anyone at this time? What do you think you learned from this experience?
c) Exploring the experience of the Age UK Later Life service, what was helpful, what could be improved? Understanding perceptions of how the service works with any other care providers.
d) Understanding what they consider to be important in later life, why, and whether the Age UK service has enabled this.

2) *Focus groups*

For stakeholder participants a focus group was held towards the end of the data collection period within each site, facilitated by 2 research team members.

The purpose was to explore stakeholder views on the Age UK Later Life service, how people experience it, and its perceived impact. The topics of the focus group were primarily determined by participants and their interactions. Topics introduced to the group for discussion included exploring processes of the service provision (patient identification, referral, service receipt, interaction with other services), perceptions of impact and service outcomes.

3) *Deliberative Panel Discussion Workshop*

The purpose of the deliberative panel discussion workshop was to draw upon the emerging findings of the case studies, to discuss the implications for implementation of similar Age UK or other services, including identifying facilitators and barriers to change, in different socio-political, cultural and economic environments. The deliberative workshop drew upon anonymised case study data, to raise specific questions using a structured format, in facilitated 'roundtable' discussions. Three discussions were held, in 2 groups, across the day. These sequentially focused on stimulus data and discussion on the following topics:

a. What is the service for? Who might benefit?
b. Concepts and challenges of the service
c. What does an ideal service look like?
